# Dr. Marshall Hall on Semeiology

**Published:** 1824-03-01

**Authors:** 


*An Essay on the Symptoms and History of Diseases; considered chiefly in their Relation to Diagnosis.* By MARSHALL HALL, M.D. F.R.S. E. and formerly Senior President of the Royal Medical Society of Edinburgh. Octavo, pp. 130. London, 1822.

OF the various parts of which medical science is composed, semeiology, the observation of symptoms or signs, is assuredly the most important. Accurate symptomatology will often make up for defective pathology—that is, for *post mortem* researches; whereas, morbid anatomy is nothing—worse than nothing—without semeiology. Many of the ancients, and even of the moderns, have made good practitioners by careful observation at the bedside, unassisted by the light of pathological anatomy; and it has been well observed by Corvisart—"de toutes les sciences physiques il n'en est peut-être pas une dans laquelle il importe plus d'interroger les sens, que dans la medecine pratique strictement dite. Toute theorie se tait ou s'évanouit presques toujours au lit des malades pour ceder la place à l'observation et à l'experience."

It is curious that, considering the great number of monographs which our continental brethren have published on semeiotics, we had none to refer to in the English language till Dr. Hall's work appeared.* The great Sprengel is the author of a large volume on this subject; and, in France, Drs. Double and Landré-Beauvais have each published extensive treatises on semeiology. The immortal Baglivi was strongly impressed with the vital importance of accurate symptomatology, as appears by the following sentence:—"*Noster in hoc opere scopus pertinet, ut dilucide cognoscatur quantum momenti in medicinâ afferat observatio.*" But as diseases, and consequently their symptoms, vary in different countries, times, and places, we cannot draw the full tide of advantage from works published at a great distance from us—consequently it becomes doubly necessary that accurate observers, in our own country, should occasionally turn their attention to semeiotics; and we are confident their time and labour would be well repaid. There are few who are better adapted for this kind of undertaking than Dr. Marshall Hall, whose accuracy of observation is only equalled by his zeal and industry. We hope, therefore, that this able physician will prosecute the subject farther, and improve the arrangement, which is not very lucid in the present form of the work. It is hardly necessary to add, that this Essay is a new and amended edition of the first part of his work on diagnosis, which is not so fully known to the profession as its merits entitle it to be. On this account we shall lay before our readers such specimens of the present performance as will probably induce our junior brethren, at least, to place the original in their library for frequent reference, as well as careful perusal.

---

* We mean that there are no works of this kind in common circulation. Some may have been published, but they are not to be seen. Thus in 1791, Dr. Price published a "Treatise on the Diagnosis and Prognosis of Diseases," but it is only to be found in the libraries of the curious.

Our author properly observes, that the immediate and ultimate objects of clinical medicine are the *identification of diseases, and the appropriation of remedies.* The sources of evidence for the *first* object are the symptoms, history, and *post mortem* examinations. The last of these sources is omitted in the present essay, which is confined to symptoms and history.

Although symptomatology and pathology should generally be studied in connexion, not separately, yet the study of the first embraces an object unconnected with the latter—namely disorder of function, which frequently leaves nothing for the scalpel to reveal after death. It is very desirable, also, but very difficult, to determine the period when functional passes into structural disease.

It has been long remarked, and sincerely regretted, that practical knowledge in medicine, can be taught but to a very limited extent, and that the precious fruits of experience necessarily die with their possessors. But this admission shews the importance of devising the means of rendering medical knowledge more capable of being imparted from one person to another. We have no doubt, but that this desirable object may be *facilitated* by presenting the young clinical student an analysis of those general impressions which constitute the object of experience in the senior. And even where knowledge cannot be transferred, the attempt is useful, because it brings into exercise the faculties, and excites the attention of the tyro.

Chapter I. *Countenance.* The fathers of physic, especially Hippocrates and Celsus, paid great attention to the countenance of the sick. "Medicus neque in tenebris, neque a capite ægri debet residere; sed illustri loco adversus eum, ut omnes notas, *ex vultu* quoque cubantis, perspiciat." *Celsus.* There are changes, induced by disease, in the surface, circulation, cellular substance, muscles, particular features, and *general expression of the face*, which the experienced practitioner turns to good account, and which the young practitioner should early learn to appreciate. It was but a few days ago, that we were invited by a young medical friend, while we were passing a door with the wrapper tied up, to go up stairs to see a patient who had but a few days, perhaps only a few hours, to live. On entering the room, the patient's face was turned to the light of a large window, and the immediate impression was, that death was *not* pourtrayed in his countenance. A minute examination of the case convinced us that the symptoms, though formidable, were by no means indicative of such imminent danger as was apprehended; and the result verified our first impression. The man is now out of danger. Unfortunately we are utterly incapable of conveying by language any idea of the traits of physiognomy, by which the conviction was impressed on the mind in this and many other instances.

The cuticular surface of the face is much affected by the state of the digestive organs. Deranged function of the liver very generally produces a sallow or yellowish tinge of the complexion. Much also depends on the state of the cutaneous *circulation,* as affecting not only the colour, but the turgidity or shrinking of the surface.

"Amongst the particular features it is of moment to observe the eye, the prolabia,—the brow, the nostrils, the lips, &c. The eye, in particular, affords the opportunity of judging of the degree in which the serum is loaded with bile in cases of icterus, and of distinguishing that disease from those morbid affections in which the complexion becomes sallow and icterode from the state of the cutis and cutaneous circulation. The state of the prolabia affords an index of other states of the blood,—as of a too serous condition, or of a defective arterialization.—The nostrils, carefully observed, denote the condition of the respiration." 16.

Dr. Hall now proceeds to point out the appearances presented by the face in particular diseases, beginning with the "*common acute fever,*" or that which usually arises from cold, fatigue, anxiety, &c. In this form, he observes, there is a diffused, vivid flushing of the countenance, frequently with turgidity, especially in the young and sanguineous. The tunica albuginea is apt to be suffused, and there is great febrile heat, with anxiety, tremor of the lips in speaking, and rapid movement of the nostrils. The tumidity diminishes as the fever runs its course.

"In the *Acute Symptomatic Fever* the countenance has a very different aspect, which it is important to observe, especially in a diagnostic point of view. The heat, turgidity, and flushing, the suffusion of the eyes, the tremor of the lips, and the *hurried* movement of the nostrils are absent, whilst the surface is frequently affected with perspiration.—There is also usually an appearance peculiar to the primary disease." 17.

We fear we do not clearly comprehend our author in this last quotation. If by "acute syptomatic fever," he means the fever resulting from acute inflammation of an organ, as the lungs, the liver, or the intestines, then our experience does not coincide with his. Surely, in the fever attending on and caused by acute pneumonia or carditis, Dr. Hall will not contend, that the heat and flushing of the face, or the hurried movements of the nostril, in embarrassed respiration, are wanting. The appearances of the face, in fact, vary much, according to the *seat* of the inflammation producing the symptomatic fever. We need only instance pneumonia and enteritis, as presenting marked differences in this respect.

"In *Chronic Symptomatic Fever* the appearances are peculiar. There is a characteristic expression of disease which strikes the common observer, and, still more, the experienced physician; the surface and complexion are cool and pale, or affected with transient or partial heat and flushing, usually without sallowness, frequently with slight lividity, sometimes with cool moisture; there are emaciation and shrinking, the cheeks falling in, the action of the muscles becoming apparent, and the skin forming into greater or smaller folds.—These appearances of the countenance are however greatly modified by the nature and seat of the original disease, as will be particularly noticed hereafter." 18.

Dr. Hall's remarks on the countenance in the different grades of typhus fever are very accurate, but need not detain us. His observations on the appearances in disorders of the digestion (denominated the *mimoses* in another work of Dr. Hall's) contain much originality, and are fully deserving of the practitioner'a attention.

"The most severe or acute form of this affection is accompanied with some paleness and sallowness, and a dark hue about the eye; the cutaneous vessels exude a little oily perspiration; the prolabia are slightly pale and livid; the muscles of the face, and especially of the chin and lips are affected with a degree of tremor, particularly on hurry or surprise, or on speaking. With this state of the countenance there are *conjoined* peculiar morbid states of the tongue, and of the hands, which will be deseribed in their proper place.

"A state of sallowness of complexion, unaccompanied with the appearances just described, usually attends the more *chronic form* of this affection, denominated *Dyspepsia.*" 21.

On *Chlorosis* many acute diagnostic remarks are made. He properly observes that the *icteroid* appearance of the complexion in chlorosis should not be confounded with *icterus* itself. In this last the tunica albuginea is tinged proportionately to the general surface.

"The term Icterus is merely expressive of a *symptom* of disease, although it is daily named and has long been arranged as a distinct *disease*. The shade varies from yellow to green or blackish. But the most important and only *practical* distinction, with regard to Icterus, is that of its *causes* or of the *primary disease;* the principal of these are 1. *constipation or loaded bowels*, 2. *acute disorder of the digestive functions*, 3. *disease of the liver*, 4. *gall stones*, 5. *hydatids in the gall ducts*, 6. *organic tumors in the abdomen, situated near the biliary ducts*, 7. *the pregnant uterus*, 8. *disease of the right kidney*, 9. *or even of the right lung or cavity of the pleura.*" 23.

In the attack of apoplexy, our author observes, there is usually, at first, a general tumidity, flushing, and lividity of the countenance, the pupils being contracted, then dilated, and often unequal. The features frequently lose their symetry, those of one side being unusually acute, while those of the other are relaxed. The whole countenance is drawn, or the expression lost in coma. The effects of paralysis on the countenance we need not describe.

In the following quotation, there is much accurate observation evinced; and in the first paragraph, the reader will remark something like want of consistency with a passage quoted a page or two back, on "*acute symptomatic fever.*"

"In *Inflammation of the Chest with acute Pain*, or *Pleuritis*, the degree of the pain is marked by a proportionate contraction of the features in general and by acuteness and elevation of the alæ nasi; the *nostrils are moved and dilated by the alternate acts of the respiration;* there is sometimes a degree of *vivid flushing*, terminating abruptly and bounded by whiteness towards the nose; the heat is inconsiderable, and there is frequently perspiration.

"In *Inflammation of the Chest with dull Pain*, or *Pneumonia*, there is less contraction of the features, but there is an appearance of anxiety, and the nostrils are widely dilated before each inspiration; there is a little heat, but frequently a degree of perspiration.

"In *Inflammation of the Chest with great Dyspnœa*, and in cases of *Effusion into the Lungs*, there is usually a general and deep suffusion of the countenance, sometimes amounting to great lividity and conjoined with turgidity, there is great anxiety, the nostrils are widely dilated on inspiration, and drawn in above the lobes; during inspiration too the pomum adami, and even the chin, are sometimes drawn downwards; the surface is cool and sometimes damp.

"The dawn of *Phthisis Pulmonalis* is marked by a delicate paleness alternated with transient gentle flushing, slight lividity of the prolabia on exposure to cold, an appearance of indisposition, frequently motion of the nostrils from the respiration, and frequently a quivering of the chin and lips on speaking.—Its progress is denoted chiefly by gradual emaciation, in addition to an aggravated state of the other morbid appearances just mentioned.

"In *Hæmoptysis* there is usually a florid state of the complexion, and frequently the effects of dyspnœa are observed in an acuteness and movement of the nostrils. If the hæmorrhagy has been very great, there may be paleness, lividity, coldness, and a clammy perspiration, with great anxiety." 27.

In most of the organic diseases of the heart, the countenance is considerably affected. "In those cases, in which the pulmonary circulation is not impeded," says our author, the complexion simply becomes unusually vivid and florid." In our own experience, which has not been very limited, we have rarely seen organic affection of the heart, without disturbance of the pulmonary function, especially on any considerable exertion of the body, or emotion of the mind.

"In *Inflammation of the Abdomen with severe Pain* there is a continued state of contraction of the muscles of the face, inducing an unnatural acuteness of the features; the forehead is wrinkled and the brows knit,—the nostrils are acute, drawn upwards, and moved by the alternate and irregular acts of the respiration,—the wrinkles which pass from the nostrils obliquely downwards are deeply marked, —the upper lip is drawn upwards and the under one, perhaps, downwards, exposing the teeth,—the chin is often marked with dimples. This state of the features is aggravated on any increase of pain—from change of position by muscular effort, or from external pressure.—Indeed in cases of abdominal affection it is better to press on the abdomen, or beg the patient to raise the head and shoulders, and *watch* the *effect* on the expression of the countenance whilst the patient's mind is occupied with some other subject, than to ask the direct question whether pressure induces pain—as is usually done; for patients naturally suppose that every *painful* part must also be *tender*, and are therefore apt to answer in the affirmative although incorrectly." 29.

Chapter II. *Attitude.* The term attitude is taken in a very comprehensive sense by Dr. Hall, embracing a consideration of *the posture and motions of the body, the state of muscular debility, power, contraction, and motion, some particular actions, and the general manner of the patient.* Our author thinks that *tremulous motions,* and the *supine posture* distinguish, in an early stage, the acute forms of idiopathic from symptomatic fevers. We should be inclined much more to trust to a minute examination of the organs and their functions, for the detection of local inflammation, than to the criterion abovementioned. It is an old, and generally speaking, a true observation, that when the patient begins to lie on his side, in febrile diseases, it is a sign of returning convalescence. It must be borne in mind, however, that in certain visceral diseases, the side is the posture always chosen by the patient. Our author makes a great many minute observations on position in fevers and other diseases, which evince his unwearied attention to every phenomenon presented to his view, and ought to serve as an example to the young—we might add—some old practitioners.

Having delineated the "posture of fever," Dr. H. goes into some instructive remarks on *tremor*, as a symptom; and these are followed by others, equally valuable, on the attitudes of patients suffering from acute dyspepsy, pulmonary consumption, affections of the head, and paralysis, whether partial or perfect. This brings us to his sections on hydrothorax and organic diseases of the heart, both of which are highly graphic; and, though we feel strong desire of transferring them to our pages, we must content ourselves with making general reference to them as interesting specimens of semeiotic discrimination and research.

The position necessary in asthma, painful abdominal inflammation, spasmodic pain of the stomach, colic, gangrene, strangulated hernia, and inflammatory disease of the kidney and bladder, are next described with much precision and conciseness. Retention of urine, in acute diseases, is often denoted by a constant elevation of the knees which is *inexplicable* until the cause be discovered.

"In *Organic Disease* in general the patient soon becomes affected with a serious, continued, and unvaried debility, stoops in walking and moves with slowness and caution. And deep-seated pain or uneasiness is often experienced from the succussion induced by *sitting down* or making *a false step in walking*, especially when there is a state of tenderness from inflammation.

"In the appearance of the *Hand* it is often easy to read a state of pain, anxiety, or other suffering,—it is closed or expanded, or variously moved.

"There is another symptom of importance to be noticed,—viz. the state of *jactitation* and *inquietude;* it occurs in different states of the system and in some diseases, but principally in cases of *irritation*, *exhaustion*, and *sinking*, and in *Diseases of the Heart*." 47.

This chapter concludes with a simple enumeration of the principal instances wherein spasmodic tremor, paralysis, contractions, convulsions, or rigidity forms the chief characteristic of the morbid state. General convulsion, we are told, p. 48, occurs in cases of diseases of the brain, especially of the parts, about its base. This is an important observation; and, we believe, its accuracy is confirmed by the best of testimony—clinical and necrotomical experience. The effect of convulsive action within the head, Dr. H. very justly observes, has not hitherto been sufficiently attended to by physicians: hysteric convulsion assumes, from long and frequent repetition, an epileptic character: epileptic convulsion often induces an apoplectic coma; and pertussis, from the violence of coughing, frequently leads to fits, and even to hydrocephalic effusion. Some acquaintance with the hysteric paroxysm has led us to regard that modification of convulsive disease as being prone to determine an excess of blood to the liver or other of the larger abdominal organs, and thus make injurious impression on the functions both of secretion and excretion. We also consider the ultimate effects of chincough as being greatly more unmanageable than the disease itself. By carrying an inordinate distribution of blood to the brain, it forms and promotes a tendency to extravasation of fluid from the lymphatics of that organ. In like manner, it predisposes to hydro-thoracic effusions, by impelling an undue proportion of blood to the heart and tissues of the chest. Practitioners can never be too vigilant in scrutinising the variations of this insidious malady.

CHAP. III. *Tongue.* The surface of this organ is affected with whiteness, load, fur, dryness, blackness, chaps; its *form* is frequently modified by its becoming swollen, indented, fissured, and lobulated; its *papillæ* are, in some cases, morbidly prominent and enlarged, and in others almost obliterated, leaving a smooth, perhaps tender, surface. It is often protruded with difficulty, from dryness, tremor, or paralysis, and is left protruded in cases of imperfect sensibility. The internal mouth, at the same time, is dry and foul; the teeth are affected with brown mucous sordes; and the breath has a peculiar odour. It is important to remark, whether with a given state of the tongue the *tendency* is to an augmentation or diminution of its morbid character.

Dr. Hall remarks, that the tongue affords one of the best diagnostics of the different kinds and degrees of idiopathic fevers, of idiopathic from symptomatic fevers, and of their complications. Its changes and tendencies denote, in a particular manner, those of the fever itself. Its diversified appearances in fever are well delineated by the author, as are those which occur in the varied forms of dyspepsy. The following is a true clinical sketch.

"The most ordinary effect of an occasional or accidental derangement in the stomach and bowels, is a loaded state of the tongue, the superior surface of this organ becoming covered with a layer of whitish, soft, mucous substance, admitting of being partially removed by the tongue scraper; the whole internal mouth is, at the same time, more or less disagreeable and clammy, the taste depraved, and the breath offensive; and frequently the substance of the tongue is a little swollen, œdematous, and marked by its pressure against the contiguous teeth. In *Acute Dyspepsia* the state of the tongue already described is observed, with some modifications:—the tongue is in general loaded, the mouth clammy, the taste bitter or nauseous, the breath fetid, whilst the surface of the face is frequently oily; in some severe cases, the *load* has been very thick, and has eventually *peeled off*, leaving the tongue red, smooth, and tender; the *substance* of the tongue is generally swollen, œdematous, and impressed by the contiguous teeth: the *gums* are often red, tumid, and somewhat separated from the teeth by tartar, and are easily made to bleed; the inside of the *cheeks*, also, frequently partakes of the œdema, and receives, like the tongue, impressions from the adjacent teeth; sometimes the cheeks and the gums of the posterior part of the mouth have been so swollen as to protrude a little over the teeth, and are either *ulcerated* by the pressure, or *wounded* by being bitten—circumstances which are apt to be induced or aggravated by cold. Through the load on the tongue, the red *papillæ* are frequently seen, either over its whole surface, or at its point principally; frequently the tongue is not only *indented*, but formed into *creases* or folds; sometimes deeper and more numerous *sulci* are formed, the edges of which are sharp and the sides in contact, and requiring to be separated by the two fingers, or by protruding the tongue further; in some cases the tongue is less loaded and indented, and its edges are red and even. In cases of the *Acute Dyspepsia*, I have seen the tongue affected with deep, foul ulcers, resulting from the slow suppuration of hardnesses about the size of a horse-bean or nut, situated just under the surface of the tongue, which is loaded, swollen, and foul, with a copious flow of saliva, and a fetid breath. In *Chronic Dyspepsia* the tongue is sometimes affected in a slighter degree, in the manner just described, being somewhat tumid, indented, and sulcated; it is in general, however, less pasty and œdematous; it is frequently covered with a sort of viscid mucus; sometimes it is slightly white from numerous, minute, white *points* crowded over its surface; it is also frequently affected with *fur*, consisting of short fibres resembling those of coarse velvet, and admitting of being separated by the finger. In this affection the tongue is frequently rather dry; and I have seen it, in several instances, sulcated longitudinally. In very *protracted* cases, the tongue assumes several remarkable modifications of form and surface:—in the *first* case there is a universal *enlargement of the papillæ* over its surface, which is now generally clean;—in two instances the papillæ at the most *posterior part* of the tongue became particularly enlarged, causing pain on swallowing, and some alarm to the patients; in the *second* modification, the surface of the tongue is formed into *lobules*, sometimes deeply intersected, and resembling in form those of the base of the cerebellum; at other times, of less regular form, and, lastly, assuming the form of squares; in the *third* variety, the tongue acquires an absolute and morbid *smoothness* of surface, which appears as if glazed, and is tense and unyielding. In all these cases, the tongue is morbidly clean, the mouth, taste, and breath being nearly natural, and its colour, although perhaps rather paler, frequently little changed; the complexion is usually rather pale and sallow, but the surface of the face is free from oiliness, and the integuments from tumidity." 54.

Chlorosis is accompanied by a very characteristic condition of the tongue. In the *beginning*, it becomes rather pallid and tumid, and has frequently enlarged papillæ over its surface; it is somewhat loaded, indented, and sulcated; the gums and prolabia are pallid; the breath tainted. At a *more advanced period*, it becomes cleaner, smoother, still more exanguious, and acquires a peculiar semi-transparency, and a very pale lilac hue. It remains a little swollen and indented, but the papillæ disappear, and often give place to a morbid smoothness. The complexion, prolabia, gums, and tongue, are alike exanguious, and perhaps a little tumid; the breath is still less tainted, and even acquires an odour of new milk; and the mouth becomes less clammy and disagreeable.

According to Dr. Hall's observation, there is an affection of the prolabium and immediately adjoining skin which he has not seen described. It consists of a repeated dry splitting and exfoliation of the cutis of these parts, and occupies a ring of about one-fourth of an inch across, all round the mouth. It varies in severity at different times, and in different cases; it is, in general, long continued, and appears to result from a protracted state of disorder of digestion.

Our author's notes on the odour of the breath, and on the mode of protruding and of withdrawing the tongue, are concise: they are evidently deduced from patient and reiterated observation.

Chap. IV. *General Surface.* The temperature of the general surface, the state of dryness, moisture, of tumidity or shrinking, or of roughness or smoothness of the skin, the colour, the occurrence of emaciation, or of œdema or anasarca, and the condition of the hands and feet, are the objects comprised in this chapter.

In febrile and exanthematous diseases the superficial temperature is, for the most part, augmented. There are others, however, in which the tendency to diminished temperature, and sensibility to cold, is great. In acute dyspepsy, this tendency prevails, and perspiration is excited by the slightest hurry or fatigue. This peculiarity is most remarkable of all, in strumous disease of the mesentery, in which the patient is greatly sensible to external cold, and to the least draught of air, and in cold weather especially, constantly draws near or hangs over the fire, until his hands and legs assume a brown colour from the influence of its heat. With this sensibility to cold, there is also frequently a great tendency to *early morning perspiration*, which appears to be induced by *sleep*, and to be in part avoided by keeping awake, which is often done purposely. In the study of *all*, but especially of *chronic* diseases, there is no point of greater importance, in our author's opinion, than that of emaciation: much might be learned by a constant attention to this subject, and when it can be done to the actual weight of patients. The degree of emaciation which takes place, depends, in part, on the nature of the *disease*, and in part on the nature and office of the *organs* affected.

There is, says Dr. H. a singular morbid affection of the surface, which has not been noticed by any practical writer, —*the face, and some parts of the surface of the body, become*

*suddenly and remarkably puffed and swollen.* This affection, in his mind, appears to be occasioned by the presence of some indigestible substance in the stomach, and generally yields to an emetic and purge. With several useful notices of certain morbid states observable in the hands and feet, this interesting chapter is concluded.

Chap. V. *General System.* The subjects discussed in this section of his Essay, Dr. H. hesitates not to say, are of an importance quite stupendous. We agree with him, and trust our readers will agree with us, in this sentiment. He cursorily refers to the various febrile manifestations, and then briefly notices other morbid states, of the general system, which he denominates those of irritation, of exhaustion, of erethismus, and of sinking. In the Doctor's opinion, the principal sources of irritation are, the presence of indigestible substances in the stomach, and especially intestinal disorder and load. He regards the effects of these, as being either gradual or sudden. Having detailed its gradual effects in another work of his,* the author wishes to direct the attention, in this place, to the *sudden* effects of intestinal irritation. These are acute pain of the head—of the side—of the loins—of the iliac region, or of some other part of the abdomen; attacks of vertigo—of dyspnœa—of palpitation—of vomiting—of hiccup; there are often great anxiety and distress—clammy perspiration—or, on the contrary, great febrile heat and its concomitant symptoms. This state is apt to be mistaken for a disease of the organ principally affected, and bleeding is injuriously prescribed, when enemata and purgative medicines are the remedies.

Exhaustion is induced by over purging, undue blood-letting, and uterine hæmorrhage, especially when they concur with intestinal irritation. Its effects may be referred to the head, heart, thoracic and abdominal viscera, and the muscular system.

"The symptoms which affect the *Head* are, severe pain; beating and throbbing; rushing, or cracking noises; vertigo, or turning round of the room, especially on raising the head or assuming the erect position; intolerance of light, and of sound; wakefulness; starting during sleep; awaking hurried and alarmed, with faintness, palpitation, feeling of sinking, of impending dissolution, &c. being overcome by noise, disturbance, or thinking even; and delirium. The

---

* "See an Essay on Disorders of the Digestive Organs and General Health, and particularly on their numerous Forms and Complications; by Marshall Hall, M.D. &c."

*Heart* is, in different cases, affected with palpitation, fluttering, irregular and feeble action; there are beating and throbbing of the carotids, and sometimes even of the abdominal aorta; a frequent, bounding, and sometimes irregular *Pulse;* faintishness or fainting, urgent demand for the smelling-bottle, fresh air, fanning, bathing of the temples; feeling of impending dissolution; incapability of bearing the erect position, and sometimes early fainting from the use of the lancet. The *Respiration* is affected in different cases, with panting, hurry, sighing, great heaving, gasping, blowing, moaning, catching, &c. and, as has been stated, with urgent demand for fresh air. There is sometimes a sense of great and alarming *oppression* about the *Chest.* There is, in some cases, an *Irritative Cough*—in violent fits—or in the form of continual hacking; this cough appears to originate in the larynx or trachea. The *Stomach* is liable to become affected with irritability, sickness, retching, vomiting, hickup, and eructation; the *Bowels* with constipation, or diarrhœa, pain, flatus, distension, &c. There are very frequently urgent restlessness, tossing about, and jactitation. In some cases, various *Spasmodic Affections* have occurred. We have often to combat the effects of exhaustion in the *puerperal state*, and in cases in which bloodletting has been improperly employed for diseases not inflammatory, or too lavishly in cases of inflammation." 75.

Dr. Hall has observed an extraordinary similarity between the symptoms of the disease described, under the name erethismus mercurialis, by Mr. J. Pearson and Dr. Bateman, and the effects of exhaustion. The descriptions of these authors do not, however, comprise the affections of the head, otherwise, he concludes, the erethismal state and that of exhaustion would be identical.

" Sinking occurs under very different circumstances and accordingly presents very dissimilar phænomena:—it occurs sometimes as a gradual and simple feebleness, decline, and cessation of the functions of circulation and respiration; at other times it takes place with the more active symptoms of inquietude and jactitation, catching respiration, hiccup, &c. sometimes it has the remarkable effect of dissolving the chain of morbid actions and sensations constituting the disease under which the patient has laboured, and of presenting to his friends, and perhaps to his unwary physician, the appearance of amendment, when life is soon to terminate in an unexpected dissolution; lastly, the appearances of sinking quickly follow the accession of gangrene." 76.

From Dr. Hall's observations, it appears that there is a remarkable similarity in the symptoms attending intestinal irritation, exhaustion from loss of blood, the erethismus mercurialis, the morbific effects of digitalis and other poisonous vegetables, sinking, and dissolution. He considers it a question of great importance, how far the existence of one of these states tends to the superinduction of another. The effects of exhaustion are apt to supervene in cases of intestinal irritation, and are, he thinks, far less liable to occur, from the same application of its causes, during the existence of internal inflammation: a given degree of intestinal irritation, on the other hand, produces unusual effects in cases of exhaustion.

Chapter VI. *The Brain.* This chapter embraces a view of the derangements observed in the energies of the brain in general, the sleep, the mental faculties and the temper, the senses and sensations, and the motions, voluntary, functional, and the sphincters. It is minute by being very free from diffuseness; concise, and at the same time comprehensive. Much practical knowledge may be derived from a careful perusal of it.

Chap. VII. *Respiration.* Under this head are described the different kinds of dyspnœa and cough,—the effects of a full inspiration and expiration,—the affections of the voice and articulation, and other errors of the respiratory function. As a specimen of the important topics discussed in it, we select the author's remarks on the effects of a full inspiration and expiration.

"A deep inspiration is *apt* to induce cough when the structure of the lungs is affected with inflammation or disease. But this morbid state is more distinctly ascertained by an attention to the effects of a *full expiration.* In many cases of morbid affection of the lungs, indeed, in which a deep inspiration induces neither cough nor other inconvenience, a full expiration not only occasions cough but other effects which vary according to the *nature* of the pulmonary disease; —in *Inflammation of the Bronchia*, or of the *Lungs*, in *Tuberculous Phthisis*, &c. cough and rattling or crepitus are induced; these effects are also induced in some cases of chronic affections of the lungs arising either from slight but protracted inflammation or from disorder of the digestive organs; in cases of *Asthma* too, in which the ordinary breathing, or a deep inspiration even, is unattended with any peculiarity, a full expiration excites both cough and the wheezing sound so characteristic of this affection. It is particularly useful to watch the efforts of a full expiration in the slighter affections of the pulmonary structure, and in the decline of pulmonary disease. Of these two modes of ascertaining disease of the lungs, I have long considered the deep expiration the most valuable but the most neglected; I therefore strongly recommend it to the attention of the clinical student." 100.

Chapter VIII. *Circulation.* Error of the circulation is observed in the pulse, in the pulsations of the heart, of the carotids, and abdominal aorta, and sometimes of the jugular vein,—and in the capillary vessels. Among many others, the following are useful hints.

"In the latter stages of inflammatory diseases, the pulse is apt to become or to remain unnaturally frequent as an effect of the *loss of blood* from repeated venæsection; it is therefore important to observe every symptom, not to be misled by a continued frequency of the pulse.

"It is not unusual to observe that in various diseases the frequency of the pulse remains, when the morbid actions have apparently subsided; in such a case it is necessary to continue our attention, and watch and wait for the diminution of the frequency of the pulse, and, if this event do not take place in a moderate space of time, to ascertain whether the disease be in fact subsided, or only mitigated and pursuing its course in an insiduous form." 103.

CHAPTER IX. *Alimentary Canal.* This division of Dr. Hall's work comprises brief notices of the symptoms which may be taken from the morbid affections of the pharynx and œsophagus, the stomach and bowels, and the sphincter ani.

We exemplify the author's manner of treating this branch of his subject, by an extract.

"In some cases both of disease of the liver and of the mesentery, but especially the latter, the appetite has been great, the food has passed off quickly, and the motions have been copious, fetid, and light-coloured. They are generally pale and fetid when the food passes through the alimentary canal rapidly, as in *Lientery.* They are very offensive in all cases attended with rapid *loss of flesh,* but in none more than those in which the powers of life are, at the same time, in a state of decline,—as in the decay of *old age,* in cases of *slow fevers,* &c. *Mucous* evacuations are the effect of irritation or inflammation of the mucous membrane of some part of the intestinal canal, especially the rectum and colon. They occur in the *Dysentery,* as the effect of *Corrosive Poison,* and in cases in which the rectum is irritated by impacted and scybalous fæces, and are often mixed with blood: in the last case there are often severe attacks of pain in the seat of the sigmoid flexure of the colon, and copious discharges of blood,—effects which are relieved by evacuating the rectum by glysters." 115.

CHAPTER X. *Urinary Organs.* The symptoms connected with morbid states of the urinary functions relate to the secretion, excretion, and condition of the urine, and to the substances which are apt to be mixed and expelled with this fluid.

When retention of urine is observed in the late stages of typhous fever, it is of very unfavourable augury. It occurs in diseases of the brain and spine, and is found as an effect of debility and of insensibility in general. It is not unusual after delivery, and is sometimes induced by the action of cantharides. It also forms a symptom in retroversion of the uterus, and of other cases in which the neck of the bladder is compressed by the pelvic organs. In the cases attended by insensibility, Dr. H. has observed a constant *elevation of the knees*, as the effect of the retention of urine, and of the distended and tender state of the badder.

In hysterical and nervous affections, the urine is copious and limpid, and remains, on cooling, free from sediment. In other cases, particularly in derangements of the digestive functions, it is so charged with matters in solution, that there is not only a sediment, but a pellicle on its surface from evaporation or exposure to the air. Dr. H. is not prepared, he says, to distinguish the different kinds of sediment, and characterise them by their proper chemical qualities, but he has observed that they are sometimes consumed, and sometimes fixed, in the fire. We should be guided, he observes, in precribing for the gravel, not by the particular *character of gravel* which has last appeared, but by the *actual disposition* of the urine to deposit a red or white sediment. At the time of writing, Dr. Hall had a patient, who discharged a qnantity of dark-coloured blood on any exposure to severe cold : the affection yielded to the genial influence of a warm bed.

Chapter XI. *Uterine System.* Dr. H's statements here, are very general : they relate to catamenial retention or suppression, menorrhagia, fluor albus, and morbid discharges. There is no distinction of these morbid discharges ; perhaps, on this subject, the doctor might have consulted with advantage, Mr. Clarke's book analysed at p. 757, Vol. II. for 1821-2, of this Journal.

We are now arrived at Part II. of Dr. Hall's Essay, and find it devoted to the *history* of diseases. It occupies about seven pages of his book, and is divided into two chapters, on their cause, their course, and the effects of remedies,—and on certain diagnostic and practical rules for dispensary practice. What the doctor has chosen to say on these subjects, will be found to constitute very useful hints for young physicians, to whose serious attention we would recommend the work altogether, as exhibiting the rudiments of Semeiology, in a manner very much distinguished by medical and philosophical observation.

